# Guideline for the diagnosis and treatment of incomplete Kawasaki disease in children in China

**DOI:** 10.1186/s12887-024-04961-2

**Published:** 2024-07-26

**Authors:** Fuyong Jiao, Yan Pan, Zhongdong Du, Fangming Deng, Xiaodong Yang, Hong Wang, Jie Shen, Wei Xiang, Zhilong Mu, Chunyan Gao, Jinmei Bai

**Affiliations:** 1 Children’s Hospital, Shaanxi Provincial People’s Hospital, Xian, 710000 China; 2 Department of Pediatrics, the First Affiliated Hospital of Yangtze University, Jingzhou, 434000 China; 3 National Center for Children’s Health, Beijing Children’s Hospital, Capital Medical University, Beijing, 100000 China; 4 Editorial Department of Chinese Journal of Contemporary Pediatrics, Xiangya Hospital, Central South University, Changsha, 410000 China; 5 Department of Cardiology, Shanghai Children’s Hospital, Shanghai Jiaotong University, Shanghai, 200120 China; 6 Department of Pediatrics, Shengjing Hospital of China Medical University, Shenyang, 110004 China; 7 Department of Cardiology, Shanghai Children’s Medical Center, Shanghai Jiao Tong University, Shanghai, 200120 China; 8 Hainan Women and Children’s Medical Center, School of Pediatrics, Hainan Medical University, Haikou, 570100 China; 9 Department of Pediatrics, the Affiliated Hospital of Yan’an University, Yan’an, 716000 China

**Keywords:** Incomplete Kawasaki disease, Clinical guideline, Randomized controlled trials, Expert recommendation, Expert panel’s guideline

## Abstract

**Background:**

Kawasaki disease (KD) is a pyretic ailment predominantly observed in children aged below 5 years. There is currently a dearth of precise markers for timely identification of incomplete Kawasaki disease (IKD). It is imperative to develop updated, comprehensive, and evidence-based guidelines to effectively direct clinical practice.

**Methods:**

The guideline development group comprised individuals with diverse expertise in both content and methodology and carried out an extensive exploration of the following digital repositories: CNKI, VIP, Wanfang Data, UpToDate, BMJ, Clinical Evidence, National Guideline Clearinghouse, Joanna Briggs Institute Library, Cochrane Library, and PubMed. The entire period from the establishment of these databases until January 1, 2024 was covered. To evaluate IKD, systematic reviews and randomised controlled trials were assessed using the risk of prejudice instrument specified in the Cochrane Handbook, along with the evidence robustness framework established by the GRADE group. The recommendations were formulated based on the findings, considering the evidence strength. After several iterations of expert consensus, the relevant professional committees in China endorsed the ultimate guideline.

**Results:**

These guidelines address clinical questions regarding the classification and definition of KD, diagnosis of IKD, treatment during the acute phase of IKD, and follow-up of IKD.

**Conclusions:**

To provide healthcare professionals with guidance and decision-making bases for the diagnosis and treatment of IKD in China, 13 recommendations were formulated based on expert consensus and evidence of best practices.

## Background

Dr. Tomisaku Kawasaki first recorded the initial clinical criteria for diagnosing Kawasaki disease (KD) in 1967, which have been utilized for a prolonged duration. KD is a self-limiting systemic inflammatory disease that occurs predominantly in children younger than 5 years of age. The incidence of KD remains the highest in Japan [1], followed by South Korea and China [2]. However, it is much lower in European and American countries. A study in Beijing indicated an increasing prevalence of incomplete Kawasaki disease (IKD) among children with KD and that there was no difference in the sex ratios of IKD and complete Kawasaki disease (CKD) [3]. Seasonal correlations have been proposed by other researchers regarding the initiation of IKD. Studies have reported that its onset occurs mostly in spring and summer in China [4, 5], whereas scholars from the United States [6] and Japan have found that its peak incidence is in winter, suggesting that seasonal differences in the onset of IKD may be associated with epidemiological changes in viral infections [7]. The onset of IKD is also believed to be influenced by patient age. Children with IKD are younger than children with CKD, with a higher percentage of children aged ≤ 2 and ≥ 6 years [8]. Approximately 75%–80% of IKD cases in American children are found in patients younger than five years (median age 1.5 years). The estimated ratio of male to female patients in children with IKD is 1.5:1 [9]. According to reports, the occurrence of IKD in children under the age of one is four times that in children older than one year. IKD in children < 6 months old was found to be 28%; of these, 85% developed coronary artery disease [10–12].

The aetiology of KD remains unclear. Based on the epidemiological and clinical characteristics of KD, the aetiology of KD may involve certain strains of the human microbiota. KD, MIS-C, and other infection-related diseases may be elicited by substances derived from infected cells, including toxins, pathogen-associated molecular patterns (PAMPs), damage-associated molecular patterns (DAMPS), and pathogenic proteins and peptides. Additionally, it is known that the microbiota differ according to age and ethnic groups and can be changed by diet and antibiotics. Thus, marked differences in the incidence of KD, MIS-C, and infection-related diseases such as juvenile idiopathic arthritis and inflammatory bowel disease across populations and different age and sex predilection can be explained by the colonisation states of pathogens derived from microbiota, not by genetic factors [13–15].

A recent theory suggests a link between tropospheric air currents and the epidemic diffusion of KD owing to the transportation of mycotoxins from China and other areas of the Asian continent [16]. In addition, a hypothetical pathogenesis of KD is proposed under the premise of a "protein homeostasis system"; where innate and adaptive immune cells control pathogenic proteins that are toxic to host cells at a molecular level. After infection with unknown KD pathogen(s), the pathogenic proteins produced from an unknown focus spread and bind to endothelial cells of the coronary arteries as the main target cells. Immune cells are activated to control the action of pathogenic proteins and/or substances on injured cells. Initially, nonspecific T cells and non-specific antibodies are involved in this reaction, while hyperactivated immune cells produce various cytokines, leading to a cytokine imbalance associated with further endothelial cell injury. After the emergence of specific T cells and specific antibodies against the pathogenic proteins, tissue injury ceases and a repair reaction begins with the immune cells [13, 17].

KD is categorised into two types: CKD and IKD. Typical KD or CKD have clear diagnostic criteria; however, certain children diagnosed with KD in the clinic do not meet the existing diagnostic criteria. Their diagnoses and clinical manifestations differ, with some symptoms appearing late or not at all, making them somewhat invisible and overlapping with the clinical symptoms of various paediatric infectious or connective tissue diseases. This increases the likelihood of misdiagnosis and underdiagnosis, leading to residual cardiac complications, and even death [18]. Currently, there is no universally accepted standard for diagnosing IKD, resulting in significant variations in reported incidence rates. A retrospective cohort study revealed that more than 25% of patients treated for IKD experienced either over or under treatment in the USA [19, 20]. IKD was found to have an incidence of 19.4% [21] and 28.4% [22] in relevant studies in China.

Although clinical research on KD has improved in recent years, the number of clinical studies remains limited. From 2021 to 2022, China successively formed an expert consensus on the application of intravenous immunoglobulin (IVIG) [23], aspirin (Asp) [24], and glucocorticoids (GC) [25] in paediatric KD, which has played an important guiding role in its clinical diagnosis, treatment, and management. However, many challenges remain in the diagnosis and treatment of IKD in clinical practice. Currently, there is a lack of evidence based on systematic evaluation and relevant recommendations (clinical practice guidelines) that balance the advantages and disadvantages of different intervention measures in the diagnosis of IKD in China. Therefore, to further standardize the diagnosis, treatment, and long-term management of paediatric IKD in China, we invite domestic experts to fully discuss and provide recommendations for the diagnosis and treatment of paediatric IKD, aiming to provide guidance for the clinical standardized management of paediatric IKD in China and reduce the occurrence of cardiovascular events in IKD.

## Methods

### Literature inclusion and exclusion criteria

The inclusion criteria were: research type, clinical practice guidelines, expert consensus, systematic reviews, and RCTs, regardless of whether a blind method is used. Object of observation: KD. The exclusion criteria were: the content of the same paper appears in both conference papers and journal papers, except for conference papers.

### Literature search

The keywords were “kawasaki disease, skin mucosal lymph node syndrome, clinical practice guidelines, expert consensus and children”. The start date was determined by the date the database was founded, and the deadline was set for 1 January 2024. The data sources included the CNKI, VIP, Wanfang, UpToDate, BMJ, Clinical Evidence, National Guideline Clearinghouse, Joanna Briggs Institute Library, Cochrane Library, and PubMed databases. The literature screening process was conducted independently by two individuals using the Noteexpress software. After completion, a third party reviewed and assessed the conflicting literature to determine its inclusion or exclusion.

### Evidence grading-GRADE standard

High: There is strong evidence that the effect estimate approximates the real effect value; Moderate: The effect estimate is moderately confident, but there is still a possibility that the real value is not identical to the estimate; Low: There is a possibility of a significant difference between the estimated value and the actual value; The confidence in the effect estimates is limited. Very low: The effect estimate is highly unreliable; the actual value differs significantly from the estimated value.

### Rules for making GRADE recommendations grading criteria

Highly recommended: The following conditions must be met simultaneously: Interventions have obvious advantages, evidence quality is high or moderate, and medical costs are low, interventions are easy to implement, operate, and the risk of side effects is low.

Weakly recommended: The following conditions must be met simultaneously: A strong recommendation for the interventions is weak because the benefits clearly outweigh the disadvantages. Medical costs are high; interventions are not available at all hospitals; Expert consensus on therapy and clinical experience without supporting modern literature evidence; Despite a poor quality level of evidence, a majority of experts agree (Table 1). The above circumstances are weakly recommended. Strongly not recommended: No Expert consensus has been reached, and the disadvantages of the interventions outweigh their advantages. Weakly not recommended: The advantages and disadvantages of the intervention are unclear, the evidence quality is low, and no expert consensus has been reached [26].

**Table 1:** GRADE quality of evidence and strength of recommendation grading

Form	Explicit description
Grading the quality of evidence	
High (A)	There is a high degree of confidence that the observed values are close to the true values
Medium (B)	Moderate confidence in the observed value: the observed value is likely to be close to the true value, but it is also likely to be very different. value, but may be very different
Low (C)	Limited grasp of observations: observations differ significantly from true values
Very low (D)	Little certainty about the observed value: the observed value can be very different from the true value
Recommended Strength Classification	
Strong (1)	Clearly show that the benefits of the intervention outweigh the harms or the harms outweigh the benefits
Weak (2)	Uncertainty about the benefits and disadvantages or evidence of comparable quality regardless of the quality of the evidence

### Methods used for establishing the guideline

The development of this guideline strictly followed the process and methodology outlined in the WHO Guideline Development Manual [27] and was reported in accordance with the Reporting Items for Practice Guidelines in Health Care [28], which meets the Institute of Medicine's definition of a clinical practice guideline [29]. These guidelines have been registered with the International Platform for Registration and Transparency of Practice Guidelines (http://www.guidelines-registry.cn/) under the number PREPARE-2023CN243. This guide solicited suggestions from over 20 paediatric experts nationwide through six rounds of online consultation, including specialists in paediatric rheumatology, paediatric cardiology, and methodology. And publicly solicits opinions from clinical doctors from multiple provinces, cities, and autonomous regions across the country through 3 KD related academic conferences to collect relevant clinical issues. Finally, consensus was reached on the recommended opinions through two rounds of expert inquiries and one guideline formation meeting. The full text of these guidelines was reviewed by external peer experts and approved by the Guideline Guidance committee [30–33]. This will be disseminated, implemented, and updated as planned. Ultimately, 84 articles were included, including 23 guidelines, 9 expert consensuses, 16 meta-analyses/systematic evaluations, 11 randomised controlled trials, and 25 observational studies.

### Updating the guideline

This guideline is planned to be updated within three to five years based on emerging relevant evidence, following the Reporting Items for Updated Clinical Guidelines: Checklist for the Reporting of Updated Guidelines (CheckUp) checklist.

### Recommendations

#### Clinical features of IKD

The pathological essence of IKD and CKD is the excessive activation of the body's immune system caused by systemic small- and medium-sized vasculitis. The laboratory indicators are almost identical. Among numerous laboratory indicators, CRP and ESR are considered to be the most effective indicators of systemic inflammation in KD [3]. Regarding the clinical characteristics of children with IKD, the main features are the high incidence of periungual desquamation around the finger (toe) and redness at the site of Bacille Calmette Guerin (BCG) inoculation during the fever and recovery period, low incidence of their symptoms of erythema and cracking of the lips, conjunctival congestion, oedema of the hands and feet, strawberry tongue, enlarged cervical lymph nodes, and high serum levels of C-reactive protein (CRP) [34, 35].

A comparative study was conducted by Japanese scholars to examine the frequency of the six primary symptoms associated with IKD and CKD. According to the results, children with IKD had a lower rate of cervical lymph node swelling at 35%, while those with CKD had a higher frequency of 65%. Additionally, the frequency of other symptoms in IKD was 75% for fever, 50% for rash, 65% for changes in the lips and mouth, 70% for changes in the ends of the limbs, and 75% for changes in the bulbar conjunctivae [25]. In addition, the probability of developing redness around the BCG vaccination site in the acute phase of IKD is higher than that in CKD. Therefore, it is important to focus on children who receive BCG injections for redness [36].

Most researchers believe that the probability of coronary artery disease is higher in IKD than in CKD [21, 37, 38]. According to the guidelines in the United States [20], if infants under 6 months of age have an unexplained fever for more than 5 days, they should undergo immediate cardiac ultrasonography to exclude coronary artery disease. This should be done even if they only have one of the usual symptoms of KD along with positive laboratory markers. A retrospective study founded that there was a greater occurrence of IKD (57.5%) in children with coronary artery disease compared to CKD (31.5%). This may be attributed to the fact that IKD commonly affects infants and young children susceptible to coronary artery disease [39, 40]. Additionally, the delayed diagnosis of IKD, caused by its atypical clinical symptoms, often results in inadequate disease management. A small number of children may also exhibit atypical clinical symptoms. A study conducted in Korea examined the factors that contribute to the age at which coronary artery lesions are diagnosed in patients with IKD. This study found that age-related factors did not play a significant role in predicting the occurrence of coronary artery lesions in children with IKD [41].

#### Diagnosis of IKD

Recommendation 1: Fever ≥ 5 days, or the fever duration could be less than 5 days at presentation, and lack of sufficient clinical standards (≤ 3 items). Abnormal echocardiography examination or related laboratory test results are abnormal. When excluding other diseases, which can be defined as IKD (high quality evidence, weak recommendation).

**Fig. 1:**
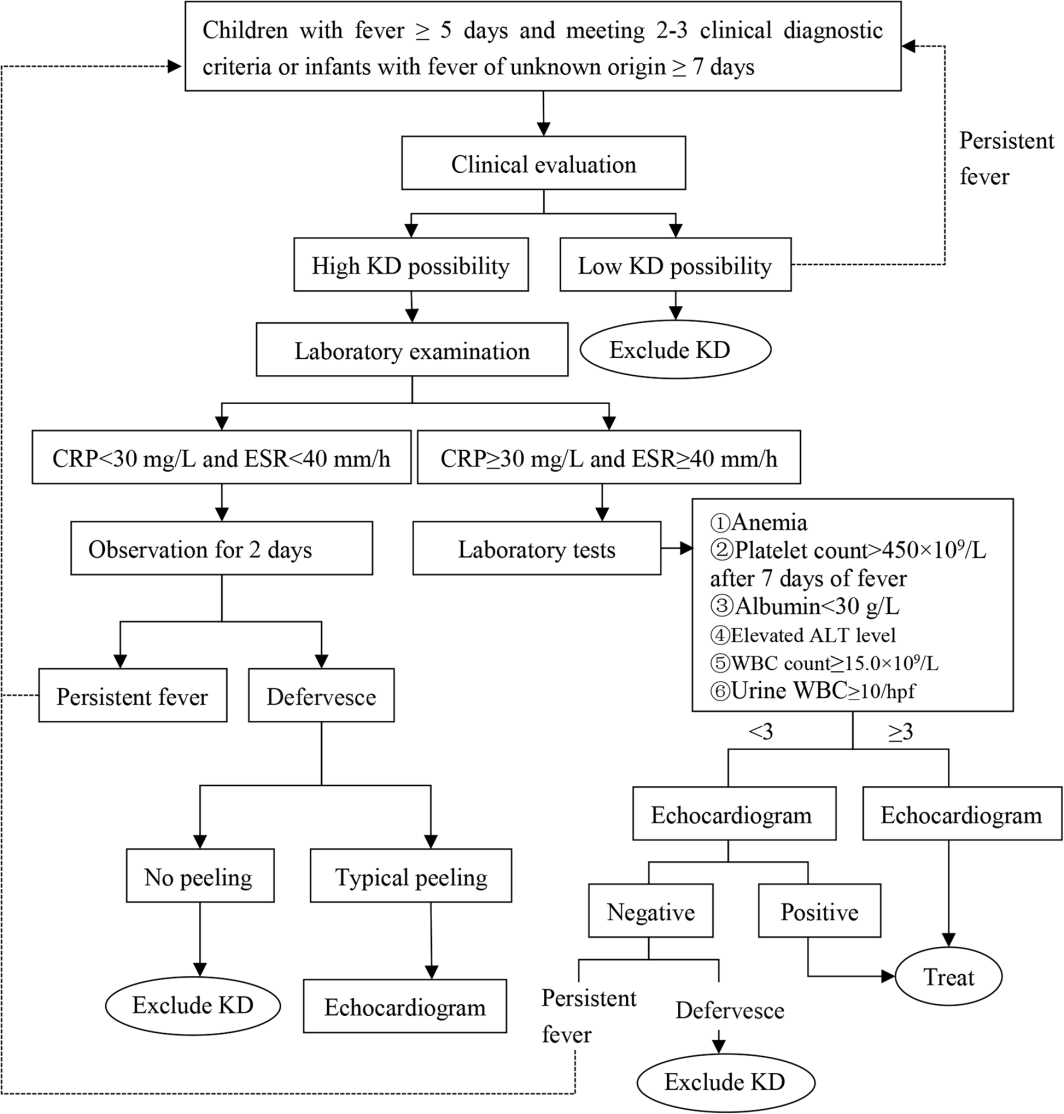
Evaluation process for suspected IKD. KD, Kawasaki disease; CRP, C-reactive protein ESR: Red blood cell sedimentation rate

Diagnosis of IKD: Fever ≥ 5 days, or the fever duration is less than 5 days, and 2 or 3 of the 5 major clinical features [(1) Non-exudative bilateral conjunctival injection; (2) Changes in lips and oral cavity, such as dry and red lips, cracking, erythema, strawberry tongue, and diffuse erythema of the oropharyngeal mucosa; (3) Acute non-suppurative cervical lymphadenopathy (usually > 1.5 cm in diameter); (4) Polymorphic rash, including erythema around BCG inoculation scars; (5) Changes in the extremities, such as palmar and plantar erythema and firm oedema of the hands and feet in the acute phase, and membranous desquamation around the nails in the recovery phase] were present or infants with fever of unknown origin ≥ 7 days, and CAL echocardiographic changes were clearly present: (1) Z value of left anterior descending coronary artery or right coronary artery ≥ 2.5; (2) coronary artery aneurysm (CAA) formation; (3) There were ≥ 3 of the following echocardiographic findings: ① left ventricular function decreased; ② mitral regurgitation; ③ pericardial effusion; ④ The Z value of any coronary artery was 2.0 ~ 2.5 [44, 47]. Or meet at least three laboratory criteria: (1) anaemia; (2) After 7 days of disease, platelet count > 450 × 109/L; (3) Plasma albumin ≤ 30 g/L; (4) Elevated alanine aminotransferase; (5) Peripheral blood leukocyte count ≥ 15 × 109/L; (6) Urine white blood cells ≥ 10/HP. In the above laboratory diagnostic indicators, if the child meets the above criteria, or it can be preliminarily diagnosed with IKD and administered standard treatment. If CRP < 30 mg/L and/or ESR < 40 mm/h in laboratory indicators and fever persists, clinical and laboratory re-evaluation is required, and echocardiographic re-evaluation is required if typical membranous peeling occurs. The assessment process of suspected IKD is shown in Fig. 1. This diagnostic process was based on American Heart Association (AHA) guidelines [20]. This guideline further optimises the IKD flowchart, emphasising dynamic observation for 2 days when laboratory tests show C-reactive protein < 30 mg/L and erythrocyte sedimentation rate < 40 mm/h.

KD is a heterogeneous syndrome consisting of mild and severe cases, such as severe CALs. Furthermore, organ involvement, such as CALs, and elevation of markers for organ involvement, such as cardiac and liver-associated enzymes, are only observed in some patients with KD. Thus, some indices, such as liver enzymes, cannot be generalised for all patients with KD. Thus, typical KD and IKD can be regarded as diseases with the same pathogenesis, similar incidence of CALs, and responsiveness to treatment. Accordingly, patients with IKD included those without CALs and those with a fever duration < 5 days. The intensity of systemic inflammation during this process is reflected in laboratory parameters, including CRP, white blood cell count (WBC) with N/L differential, albumin, haemoglobin, platelets, immunoglobulins, and other cytokines and chemokines. Furthermore, platelets, total protein, and levels of immunoglobulins (IgG, IgM, and IgA) begin to increase after the peak stage (6–7 days after fever onset) and markedly increase at the early convalescent stage [42]. Thus, the platelet level in KD is an important cue for evaluation in the acute stage of KD; for example, persistent febrile KD with decreased or non-increased platelets means that this KD case does not reach the peak stage, and follow-up platelet levels are crucial for KD selection. Long-term aspirin therapy for all KD (6–8 weeks) or KD with mild CALs, except for round giant aneurysms, may be unnecessary, since thrombocytosis may be involved in the recovery reaction in KD, including vasculitis, and mild CALs naturally subside over time [43]. The researchers could select patients with KD and IKD among the suspected subjects: those with initial CRP ≥ 30 mg/L and ESR ≥ 40 mm/h, and increases platelet level at follow-up examination at least 3–4 days apart, markedly increased platelet especially in infants.

Most febrile viral infections do not show CRP > 30 mg/L or ESR > 40 mm/h, and most febrile bacterial infections respond to antibiotics. Platelet levels > 450 × 109/L after 7 days of fever suggest that they begin to increase at the peak stage of KD, but some severely affected KD cases can be elevated after 7 days. Albumin levels < 30 g/L may be too low to detect KD; however, most patients with KD have lower albumin levels than age-matched healthy children. In the early presentation of KD, haemoglobin, albumin, and CRP levels change the day before the peak stage of KD, which is helpful for early diagnosis [44].

Early diagnosis of IKD is challenging due to the absence of a consistent pattern or standardisation in the onset of the disease, significant variations among children, and incomplete manifestation of clinical symptoms during the initial phase. Precautions for diagnosing IKD: IKD has fewer clinical symptoms and signs, and its diagnosis needs to be combined with laboratory indices and echocardiography to make a comprehensive judgement, and sometimes it even needs to be observed dynamically during the course of the disease to make a diagnosis. In the event of a negative echocardiogram for the child, it is necessary to monitor the child's temperature fluctuations. If the child no longer experiences a fever, KD can be ruled out. However, if a child continues to have persistent fever, it is advisable to recheck the echocardiogram and seek consultation from a KD specialist to determine the subsequent steps for diagnosis and treatment, with a special focus on alterations in laboratory values in children with KD who experience symptoms beyond the typical age of onset. These changes may include heightened levels of white blood cells, primarily neutrophils; reduced levels of haemoglobin; elevated platelet counts; aseptic pyuria; increased levels of C-reactive protein; heightened erythrocyte sedimentation rate; raised liver enzymes and total bilirubin; diminished albumin and sodium. In addition, elevated levels of interleukin (IL)6, IL-1, and tumour necrosis factor alpha have been observed. Insufficient validation studies have been conducted to support the use of elevated plasma brain natriuretic peptide (B-type natriuretic peptide, BNP) or N-terminal pro-B-type natriuretic peptide (N-terminal pro-B-type natriuretic peptide, NT-proBNP) as a reliable indicator for diagnosing IKD [45]. Echocardiography is of great value in the diagnosis of IKD, and examination should be performed with careful observation of the presence of coronary artery lesions (CAL), evaluation of myocardial function, valvular regurgitation, and pericardial effusion.

#### Treatment of IKD

The general approach for treating IKD is similar to that for CKD, and both recommend administering a single high dose (2 g/kg) of IVIG intravenously within 10–24 h within 10 days of disease onset. This should be accompanied by the oral administration of aspirin (30–50 mg/kg), which can quickly and effectively relieve symptoms, lower cytokine levels in the bloodstream, and decrease the incidence of CALs and CAAs [46]. Notably, IKD is easily delayed until 10 days after disease onset before diagnosis, and the absence of fever and other acute manifestations at the time of diagnosis makes it difficult to decide whether remedial treatment should be administered. Furthermore, certain young patients with IKD may experience CAA complications as a result of delayed therapy or an inadequate IVIG response.

It is generally accepted that if the child is still within 14 days of the disease, high-dose IVIG and aspirin should be administered as long as the child has a systemic inflammatory response such as reddening of the lips, conjunctival congestion, leucocytosis, CRP positivity, or increased ESR, regardless of whether the child is still febrile [23, 24]. If the child has been ill for more than 14 days and inflammatory symptoms and laboratory changes are no longer present, only low-dose aspirin (3–5 mg/kg/d) should be administered to fight platelet aggregation and prevent thrombosis, but echocardiography should be performed at the time of diagnosis and at regular intervals within the next 2 months to follow up on coronary arteries to decide on the duration of aspirin therapy [24].

The main goal of the acute phase treatment of IKD is to suppress the inflammatory response and minimise the risk of developing CAA. In practice, this means rapid suppression of the acute phase inflammatory response caused by IKD. Except for very mild IKD, IVIG should be initiated before day 7 of disease onset, and histopathological examinations have indicated that arteritis typically manifests approximately 8 to 9 days following the initiation of KD. Therefore, treatment should be initiated prior to this to suppress arteritis and accelerate the resolution of fever and normalisation of inflammatory markers.

#### IVIG

Recommendation 2: The optimal timing is 5–10 days after the onset of illness, best within 7 days (high quality evidence, strongly recommended).

KD is a self-limiting disease regardless of severity, and earlier studies in the pre-IVIG era, including Dr. Kawasaki’s historic report of 50 cases, reported that mean fever duration of KD was 10–11 days, although some severely affected patients with KD show more prolonged fever duration with/without CALs. Thus, it is proposed that the intensity of systemic inflammation gradually increases and reaches a peak (mean at 6th febrile day), and then gradually decreases and enters the convalescent stage during the febrile stage of KD [44, 47]. These findings suggest that the immune reaction of the host before the peak of inflammation may be responsible for tissue cell injury and that the immune reaction after the peak of inflammation may be responsible for tissue cell repair. Moreover, CALs begin to appear before the peak stage, and early control of inflammation before the peak stage is crucial for reducing tissue cell injury, including CALs, and morbidity [16]. Thus, patients with KD should receive immunomodulators such as IVIG or corticosteroids (CS) as soon as possible before the peak stage (within 6 days) to reduce morbidity and possibly prevent CALs. A retrospective study in Beijing showed that IVIG administration on Day 5–9 seems to be the best time for IVIG therapy in KD [48]. Guidelines of the Italian Society of Paediatrics recommend that IVIG be administered at a dose of 2 g/kg of body weight in a single infusion within the first 7th day of illness, as in the 8–9th day CAA might appear [49].

Recommendation 3: Use within 5 days after the onset of illness may lead to an increased incidence of IVIG resistance (high quality evidence, strongly recommended). Severe conditions, such as combined hypotension, shock, haemodynamically unstable myocarditis, and paralytic intestinal obstruction, should still be treated in a timely manner (high quality evidence, strongly recommended).

Because the immunopathogenesis of CALs in KD may be the same regardless of CAL severity, the progression and prognosis of CALs are dependent on the host immune system against insults from KD. Predicting the progression of CALs is difficult. Thus, clinicians should focus on large CALs and their progression by CALs with serial echocardiography. Unfortunately, immune modulators such as IVIG and corticosteroids may have a limited effect on preformed CALs and prevent their progression after defervescence, especially in giant aneurysms.

Recommendation 4: Children who have been diagnosed for > 10 days, excluding persistent fever caused by other reasons and accompanied by elevated ESR, CRP, or elevated inflammatory markers combined with CAL, still need to receive IVIG treatment (medium quality evidence, strong recommendation).

Timely application of IVIG can reduce the severity of clinical symptoms and reduce or even avoid cardiovascular sequelae in children with Kawasaki disease. A Japanese study found that the incidence of CAL was significantly increased in patients who started IVIG treatment after 11 to 20 days, compared with those who applied IVIG 4 to 8 days after onset (27% versus 1%) [46, 50, 51]. Administering IVIG treatment within the first four days after onset did not decrease the risk of CAL when compared with conventional treatment given between five and seven days after onset [52]. The optimal time to apply IVIG is within 7 days of onset, although applying it within 5 days may enhance the likelihood of IVIG reused [53]. A systematic review demonstrated that administering IVIG within 5 days of symptom onset increased the likelihood of non-response to IVIG treatment [54]. The Italian Society of Paediatrics recommends that IVIG treatment within 5 days be limited to exceptional cases with a complete and definitive diagnosis of KD [49]. The progression of KD to a severe disease, which can be combined with hypotension, shock, haemodynamically unstable myocarditis, and paralytic intestinal obstruction, is associated with the presence of higher levels of inflammatory factors and a hyperimmune inflammatory response, causing a decrease in peripheral vascular resistance, cardiac insufficiency, and capillary leakage, which should be treated with anti-inflammatory therapy as early as possible [55]. In KD, the aim of IVIG is to suppress the inflammatory reaction, counteract inflammatory agents, and safeguard the coronary arteries. Studies have shown that approximately 50% of patients with a disease duration of more than 10 days still develop CAL when IVIG is applied, but the incidence is less than that of the group without IVIG [51]. For children with more than 10 days of disease, even if the temperature is normal but inflammatory indicators are still high and accompanied by CAL, IVIG is still necessary [37].

Recommendation 5: A single dose of IVIG (2 g/kg) is usually administered intravenously within 12–24 h (high quality evidence, weakly recommended).

It is recommended to start with an initial infusion rate of 0.01 mL/(kg.min) [5% IVIG 30 mg/(kg.h)] for a duration of 15–30 min. This rate can be increased to 0.02 mL/(kg.min) and further adjusted to 0.04 mL/(kg.min) if it is well tolerated. The maximum rate should not exceed 0.08 mL/(kg.min). In Japan, the typical method of IVIG administration is intravenous infusion over a period of 12–24 h, whereas in the United States, it is usually given over 18–24 h.

Some patients experience a high-grade fever episode due to hypersensitivity reactions during IVIG infusion; these patients may have reduced CRP and WBC counts after IVIG infusion with fever episodes. To avoid this reaction, some researchers have used premedication such as antihistamines and/or hydrocortisone before IVIG infusion [56].

Recommendation 6: In patients with IKD, IVIG therapy is strongly recommended immediately at diagnosis rather than delay until 10 days or later (high quality evidence, strongly recommended).

For individuals with IKD, it is crucial to promptly commence IVIG treatment following the diagnosis of KD, particularly in the presence of fever. Approximately 80% of instances require a decrease in temperature to ≤ 37.5 °C within 48 h of commencing IVIG treatment. In 40% of patients who do not respond to IVIG, an additional 1 g/kg of IVIG can bring down the fever to ≤ 37.5 °C. If the fever persists for 48 h after starting IVIG, it should be regarded as a sign of IVIG-resistant KD. Prevention of CAA in such patients may depend largely on the choice of subsequent therapy. Echocardiography for coronary artery measurements should be performed immediately in patients with suspected IKD and fever (highly recommended, very low evidence). Patients with IKD have the same or higher risk of developing coronary aneurysms compared to patients with CKD. Echocardiography can help establish the diagnosis and start treatment quickly, possibly preventing adverse outcomes. The effect of immune modulators is dependent on the severity of systemic inflammation in KD and the immune status of the host [13]. The severity of systemic inflammation in KD is reflected in laboratory values, including CRP, neutrophil differential, albumin, and haemoglobin. Observing changes in these laboratory parameters through repeated examinations prior to the peak of inflammation in acute KD may aid in diagnosing early-presenting patients with KD [57].

Recommendation 7: For non-responsive KD (IVIG-resistant KD) with persistent or recurrent fever of any degree between 36 h and 2 weeks after the initial IVIG treatment, it should be reapplied as soon as possible, at a dose of 2 g/kg, intravenously infused within 12 to 24 h. GC or Infliximab can also be used in combination with IVIG for treatment (medium quality evidence, strong recommendation).

Reasonably, high doses of IVIG are the most effective anti-inflammatory treatment to reduce the risk of CAA in patients with KD. IVIG is derived from human plasma and informed consent is required prior to its use. IVIG should also be administered to children who develop the following symptoms after the 10th day of illness: persistent fever, no longer fever but with an aneurysm, and persistent systemic inflammation, as indicated by elevated CRP. For patients with acute KD who are at a high risk of IVIG resistance or developing CAA, it is conditionally recommended to use IVIG along with GC as first-line therapy. Infliximab treatment may be more effective than GC and second-dose IVIG treatment in children resistant to IVIG, but there was no significant difference among the three treatment methods in preventing CAA [58].

The mean fever duration at admission of KD in Western countries, and possibly in China, may be far longer than that for patients with KD in Japan and Korea. In Korea, > 50% of patients with KD are admitted within 5 days of fever onset. The levels of WBC count with neutrophil and lymphocyte differential, haemoglobin, and possibly albumin are quite different according to age in childhood, and they are also influenced by fever duration at presentation in the early stages of KD and infused IVIG. Thus, some parameters are influenced by the composition of patients of different ages, different fever durations at presentation, and organ involvement in each study; accordingly, the scoring systems for the prediction of severe cases and the risk factors for CALs, including CRP and WBC, have failed to be effective across populations and even the same nation in Japan, Korea, and possibly in China [44]. Since the majority of KD studies in China might have a mean pre-admission fever duration > 7–8 days (after the peak inflammation stage) and late-presenting patients with KD might show less severe laboratory findings such as lower CRP and higher platelet counts, and possibly more CALs compared with the results from early presenting KD reports.

Japanese risk assessment tools have been created to identify individuals with a high likelihood of IVIG resistance, however, these tools exhibit limited sensitivity and specificity when applied to the diverse American population. A recent examination of patients from North America discovered that the subsequent demographic and clinical traits can forecast the occurrence of CAA development within 2 to 8 weeks: left anterior descending or right coronary artery Z score ≥ 2.0, age below 6 months, Asian heritage, and C-reactive protein level of 13 mg/dL or higher. In the United States, the characteristics that indicate a higher probability of Kawasaki disease onset are diagnosis at an age below 6 months or above approximately 9 years.

Emerging data suggest that the addition of GC upfront with IVIG may decrease the risk of CAA. GC may potentially decrease the advancement of CAA when administered to patients upon diagnosis. Hence, the combination of GC and IVIG presents a viable treatment choice for individuals with a heightened susceptibility to CAA. The optimal dosage and duration of GC still need to be determined and further research is needed in the population, but the typical dosing of prednisone starts from 2 mg/kg/day (maximum 60 mg/day) and gradually decreasing within 15 days. When the treating physician is unsure if the patient is at high risk of developing CAA, or when additional exposure to GC may be harmful to the patient, it is conditionally recommended to use IVIG alone. The expert group in this guideline defines high-risk features of KD as having a Z-value of ≥ 2.5 in the left anterior descending branch or right coronary artery during the first echocardiography and an age of < 6 months. This definition uses a Z value of 2.5 instead of 2.0, as a Z value of 2.5 is used defined as a true aneurysm. Therefore, patients without high-risk characteristics should not receive GC.

#### Aspirin (Asp)

Recommendation 8: Asp is strongly recommended for patients with acute IKD (high quality evidence, strongly recommended).

A standard treatment for patients with IKD is the use of aspirin as an antiplatelet agent for reducing inflammation and preventing thrombosis. However, the optimal dose is unknown. In 2004, the American Heart Association recommended the use of high-dose Asp [80–100 mg/(kg·d)] orally in four doses during the acute phase of KD in its diagnostic and treatment guidelines. However, considering that large doses of Asp will cause strong gastrointestinal adverse reactions, it is recommended to use medium doses of Asp [30–50 mg/(kg·d)] in Japan and South Korea in 2–3 oral doses [59]. The recommended treatment for KD in the acute phase in China is mainly high-dose IVIG combined with moderate dose Asp [30–50 mg/(kg·d)] [25].

Recommendation 9: Asp 30 ~ 50 mg∕(kg.d) should be given orally in 2 ~ 3 doses in the acute phase of IKD, and then change to 3 ~ 5 mg∕(kg.d) after the fever subsides for 48 ~ 72 h or after the onset of the disease for 14 days. Continuous oral administration for 6–8 weeks. Children with CAL should take oral medication until the coronary artery returns to normal (high quality evidence, strongly recommended).

Recommendation 10: Children with undiagnosed KD and/or atypical KD before using IVIG can usually take Asp 3–5 mg/(kg.d) orally for 6–8 weeks. Children with CAL should take oral medication until the coronary artery returns to normal (moderate quality evidence, weak recommendation).

Patients diagnosed with acute KD who experienced resolution of fever continued to undergo daily temperature monitoring at home. Patients with KD may experience disease recurrence or refractory disease, with recurrent fever and other symptoms as precursors, and fever duration is a predictive factor for CAA. Therefore, patients should monitor their fever daily for 1–2 weeks after discharge. Fever is defined as oral temperature in older children and rectal temperature in infants > 38.0 ℃ or axillary temperature > 37.5 ℃. The physician who discharges the patient should guide parents or guardians on how to measure body temperature and inform them to contact the doctor if they experience fever again. Fever monitoring is performed daily as it is inexpensive, harmless, and can detect recurrent KD.

IKD has different mechanisms of action depending on its dosage. It exerts a strong anti-inflammatory effect in the acute febrile phase, while in the subacute phase with an increased risk of CAA, it exhibits a milder antiplatelet effect at a lower dose. During the initial stage, it is necessary to administer Asp three times a day, with a dosage of 30–50 mg/kg, until the fever subsides for a period of 48 h. Initiate low-dose aspirin (3–5 mg/kg/d) upon discontinuation of high-dose aspirin. Low-dose Asp is stopped 8 weeks after IKD initiation in patients without CAA. In children with CAA, low-dose Asp can be continued until the vascular lesions disappear or indefinitely if the vascular lesions persist. Younger than 12 months old high-risk patients with an elevated CRP, elevated aminotransferase levels, hypoproteinaemia, and severe anaemia at the beginning of the disease may exhibit early CAA development, macrophage activation syndrome, or signs of shock. Patients at a heightened risk for KD should be administered primary therapy consisting of IVIG in addition to Asp and GC.

#### GC

Recommendation 11: IVIG in combination with GC as first-line therapy: IVIG non-responsive KD; KD children with CAA or peripheral haemangioma and continuously elevated inflammatory markers. Recommended dosage and course of treatment: administer medication doses consistently, such as prednisone [1–2 mg/kg/day taken once in the morning, maximum dose not to exceed 60 mg/day] or methylprednisolone [1–2 mg/kg/day administered intravenously, either once daily or divided BID]. After body temperature and CRP are normal, oral prednisone [1–2 mg/(kg·d) was taken once in the morning] and start reducing the dosage. Gradually reduce the dosage within 15 days [1–2 mg/(kg·d), 5 days; 0.5 ~ 1 mg/(kg·d), 5 days; 0.25 ~ 0.5 mg/(kg·d), 5 days] (high quality evidence, strongly recommended).

The activation of the systemic immune system is crucial in the development of the acute phase of KD. GC suppresses the immune response and has a strong anti-inflammatory effect. Given the pathogenesis of KD, it is logical to assume that the judicious utilisation of GC could be advantageous in managing KD, alongside IVIG administered intravenously and aspirin therapy [33]. In 1979, Kato et al. [34] demonstrated a correlation between GC usage and a heightened susceptibility to CAA. Since then, the use of GC in KD treatment has been limited. However, the number of patients treated with GC in that study was small, their methodology was flawed, the subgroups were biased, and they did not differentiate between patients with KD at high risk of CAA and those who did not respond to IVIG. Therefore, the reliability of the conclusion is low. Further research has indicated that administering GC treatment during the initial stage of KD decreases the occurrence of CAA, the length of clinical symptoms (such as fever and rash), CRP, and ESR in comparison to IVIG. The reduction in the time taken for laboratory parameters to return to normal and the length of hospital stay did not increase the occurrence of CAA or serious adverse events, as reported in studies [24, 60, 61].

Recommendation 12: The second dose of IVIG or IVIG combined with prednisone (or methylprednisolone) can be chosen as the second-line treatment for IVIG unresponsive KD. Recommended dosage: prednisone [1–2 mg/(kg·d) taken once in the morning, with a total dose of < 60 mg/d] or methylprednisolone [1–2 mg/(kg·d) administered intravenously once to twice a day], gradually decreasing after normal body temperature and CRP, and then gradually decreasing within 15 days [1–2 mg/(kg·d) for 5 days; 0.5–1 mg/(kg·d) for 5 days; 0.25–0.5 mg/(kg·d) for 5 days (high quality evidence, strongly recommended).

The results of a national prospective surveillance study in Switzerland in 2023 showed that children with IKD were more frequently treated with GC than those with CKD (p = 0.01), which may be due to the higher rate of IVIG resistance in patients with IKD and the need for second-line therapy in addition to the second dose of IVIG, which seems to prevent CAA in these patients [62]. According to a previous study, high-dose methylprednisolone pulse therapy (10 or more mg/kg for 3 days) may be as effective as IVIG [58]. Thus, researchers in China could continue to study this issue to prepare for the possibility of an IVIG shortage or other situations.

Recommendation 13: GC is not recommended as a routine first-line treatment for KD (high quality evidence, strong recommendation).

GC was utilized as the primary therapy for KD before the initial report of IVIG use for KD by Furusho et al. [63] in 1984. However, it was discontinued because of the notable increase in coronary artery damage, and there is now a consensus in China that the use of GC alone as a first-line treatment for KD is unsafe and contraindicated [25]. When treating KD in children with GC alone, special attention should be paid to the prevention of Cushing syndrome, infection, thrombosis, osteoporosis, aseptic necrosis of the femoral head, diabetes, hypertension, hormonal glaucoma, cataracts, bradycardia, secondary adrenocortical insufficiency, and growth retardation. For the prevention and treatment of osteoporosis, it is recommended to supplement vitamin D 600–800 U/D and calcium 1000–1200 mg/D during GC treatment. Before high-dose methylprednisolone pulse therapy, all kinds of infections should be fully excluded, especially tuberculosis, fungi, and chickenpox. Blood pressure, blood glucose, and other indicators should be closely monitored, and timely detection and active management of these complications should be ensured. To improve the prognosis of children with KD, we should attempt to minimise adverse reactions while using GC [39].

## Limitations

The guidelines primarily consisted of previous guidelines, expert consensus, meta-analyses/systematic evaluations, RCTs, and observational studies. These recommendations are justified because they rely on high-level studies and expert consensus. Nevertheless, further rigorous trials are necessary to determine the effectiveness of treatment for IKD in the future.

## Conclusion

To summarise, we offer suggestions to aid Chinese paediatricians in the management of KD. As new treatment strategies for this illness are discovered, these guidelines can function as a valuable source for fundamental management principles and will continue to develop.

## Data Availability

The datasets utilized and analyzed in this study can be obtained upon request from the corresponding author.
